# Uma Iniciativa Nacional de Melhoria da Qualidade em Cardiologia: O Programa de Boas Práticas em Cardiologia no Brasil

**DOI:** 10.36660/abc.20230375

**Published:** 2023-11-23

**Authors:** Fabio Papa Taniguchi, Sabrina Bernardez-Pereira, Antônio Luiz Pinho Ribeiro, Louise Morgan, Anne B. Curtis, Kathryn Taubert, Denilson Campos de Albuquerque, Sidney C Smith, Angêlo Amato Vincenzo de Paola

**Affiliations:** 1 Hospital do Coração São Paulo SP Brasil Hospital do Coração, São Paulo, SP – Brasil; 2 Universidade Federal de Minas Gerais Belo Horizonte MG Brasil Universidade Federal de Minas Gerais, Belo Horizonte, MG – Brasil; 3 American Heart Association Inc Dallas Texas EUA American Heart Association Inc, Dallas, Texas – EUA; 4 University at Buffalo Buffalo New York EUA University at Buffalo – The State University of New York, Buffalo, New York – EUA; 5 American Heart Association Basel Suíça American Heart Association, Basel – Suíça; 6 Universidade do Estado do Rio de Janeiro Rio de Janeiro RJ Brasil Universidade do Estado do Rio de Janeiro, Rio de Janeiro, RJ – Brasil; 7 University of North Carolina at Chapel Hill Chapel Hill North Carolina EUA University of North Carolina at Chapel Hill, Chapel Hill, North Carolina – EUA; 8 Escola Paulista de Medicina Universidade Federal de São Paulo São Paulo SP Brasil Escola Paulista de Medicina da Universidade Federal de São Paulo – UNIFESP, São Paulo, SP – Brasil

**Keywords:** Cardiologia, Melhoria de Qualidade, Prática Clínica Baseada em Evidência

## Abstract

**Fundamento:**

Apesar de progresso significativo na melhoria da qualidade do tratamento de doenças cardiovasculares, lacunas persistem em termos de falha na adesão às recomendações de diretrizes.

**Objetivo:**

Este estudo avalia os efeitos da implementação de um programa de melhoria da qualidade adaptado do Programa *Get with the guidelines®* da *American Heart Association* sobre a adesão às diretrizes para síndrome coronária aguda (SCA), fibrilação atrial (FA) e insuficiência cardíaca (IC).

**Métodos:**

Avaliamos dados demográficos, medidas de qualidade, e desfechos em curto prazo em pacientes com SCA, FA, e IC incluídos no programa Boas Práticas em Cardiologia (BPC) entre 2016 e 2022.

**Resultados:**

Este estudo incluiu 12167 pacientes em 19 hospitais no Brasil. A idade média foi 62,5 [53,8-71] anos, 61,1% eram do sexo masculino, 68,7% apresentaram hipertensão, 32% diabetes mellitus, e 24,1% dislipidemia. Os escores médios compostos tiveram desempenho sustentável entre o período inicial e o último trimestre do seguimento: 65,8±36,2% a 73± 31,2% para FA (p=0,024); 81,0± 23,6% a 89,9 ± 19,3% para IC (p<0,001), e de 88,0 ± 19,1 a 91,2 ± 14,9 para SCA (p<0,001).

**Conclusões:**

O programa BPC é um programa de melhoria de qualidade no Brasil, em que dados em tempo real, obtidos usando métricas de diretrizes de cardiologia, foram implementados, resultando em uma melhora global no manejo da FA, IC e SCA.

**Figura Central:**
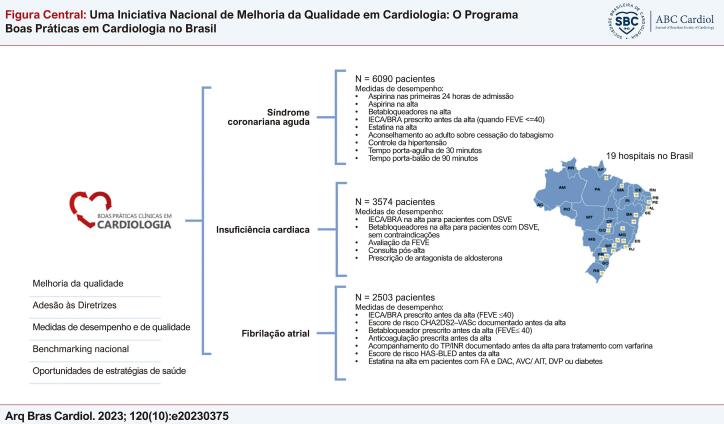
: Uma Iniciativa Nacional de Melhoria da Qualidade em Cardiologia: O Programa Boas Práticas em Cardiologia no Brasil

*IECA/BRA: inibidor de enzima conversora de angiotensina / bloqueador de receptor de angiotensina; TP/INR: tempo de protrombina/ razão normalizada internacional; FEVE: fração de ejeção do ventrículo esquerdo; DSVE: disfunção sistólica do ventrículo esquerdo; AVC: acidente vascular cerebral; AIT: ataque isquêmico transitório; DVP: doença vascular periférica.*

## Introdução

A doença cardiovascular (DCV) impõe um peso econômico e de saúde significativo no Brasil, sendo que o país apresenta uma das taxas de mortalidade mais altas por DCV no mundo, comparável com a China e o Leste Europeu.^1^ Para abordar essa questão, o programa Boas Práticas em Cardiologia (BPC) foi implementado no Brasil. O programa é liderado de maneira colaborativa pelo Hospital do Coração (HCor), pela Sociedade Brasileira de Cardiologia (SBC), pela *American Heart Association* (AHA), e pelo Ministério da Saúde, e visa melhorar o tratamento da DCV adaptando programas de melhoria da qualidade do programa *Get With The Guidelines*®.^2-6^

O programa BPC no Brasil é o primeiro exemplo em que três programas de qualidade, adaptados da AHA, são iniciados simultaneamente fora dos Estados Unidos. Especificamente, o programa visa a melhora do cuidado da síndrome coronária aguda (SCA), da insuficiência cardíaca (IC), e da fibrilação atrial (FA). Seus objetivos incluem: (1) descrever as características, o tratamento hospitalar e os desfechos de pacientes admitidos em hospitais públicos no Brasil, (2) avaliar a efetividade dos programas de melhoria da qualidade no tratamento e nos desfechos, e (3) explorar e otimizar as estratégias de melhoria da qualidade no sistema de saúde brasileiro. Este artigo apresenta os desfechos e os resultados do programa BPC, com o objetivo de identificar outras oportunidades para melhoria da qualidade e orientar o desenvolvimento de estratégias e ferramentas efetivas para melhorar os desfechos cardiovasculares no Brasil.

## Métodos

### Delineamento do estudo

O programa BPC é uma iniciativa de melhoria da qualidade com um registro nacional com foco nas medidas da qualidade do tratamento da SCA, IC e FA (Figura Central). Um procedimento padrão foi usado durante a coleta de dados dos prontuários médicos dos pacientes, e auditorias regulares de qualidade para assegurar a acurácia e completude dos dados da pesquisa foram realizadas pelo centro coordenador. O estudo foi aprovado pelos comitês de ética em pesquisa do HCor, São Paulo, Brasil (48561715.5.1001.0060) e de cada hospital participante. Detalhes do delineamento e da metodologia do programa BPC foram descritos em outro estudo.^2^

Os dados coletados incluíram variáveis demográficas, informações pré-hospitalares, diagnóstico e tratamento na internação atual, tratamento medicamentoso, eventos clínicos durante a internação, orientação de alta, e diagnóstico de alta.

### Medidas de resultados

As medidas de desempenho foram delineadas para avaliar a qualidade do tratamento de pacientes com SCA, IC e FA. As medidas foram desenvolvidas de acordo com as diretrizes da Sociedade Brasileira de Cardiologia e do *American College of Cardiology /American Heart Association*. Medidas de desempenho para cada condição crítica foram analisadas para cada centro antes e após a participação no programa BPC (Tabela 1).

**Tabela 1:** – Medidas de desempenho

Fibrilação atrial
IECA/BRA prescrito antes da alta (FEVE ≤40)
Escore de risco CHA2DS2–VASc documentado antes da alta
Betabloqueador prescrito antes da alta (FEVE≤ 40)
Anticoagulação prescrita antes da alta
Acompanhamento do TP/INR documentado antes da alta para tratamento com varfarina
Escore de risco HAS-BLED antes da alta
Estatina na alta em pacientes com FA e DAC, AVC/ AIT, DVP ou diabetes
**Síndrome coronária aguda**
Aspirina nas 24 horas de internação
Aspirina na alta
Betabloqueadores na alta
IECA/BRA prescrito antes da alta (FEVE ≤40)
Estatina na alta
Aconselhamento ao adulto para cessação do tabagismo
Controle da hipertensão
Tempo porta-agulha de 30 minutos
Tempo porta-balão de 90 minutos
**Insuficiência cardíaca**
IECA/BRA na alta para pacientes com DSVE
Betabloqueadores na alta para pacientes com DSVE, sem contraindicações
Avaliação da FEVE
Consulta pós-alta
Prescrição de antagonista de aldosterona

*IECA/BRA: Inibidor de enzima conversora de angiotensina / bloqueador de receptor de angiotensina; GWTG-HF: Get With The Guidelines-Heart Failure; FEVE: fração de ejeção do ventrículo esquerdo; DSVE: disfunção sistólica do ventrículo esquerdo; AVC: acidente vascular cerebral; AIT: ataque isquêmico transitório; DVP: doença vascular periférica; FA: fibrilação atrial; DAC: doença arterial coronariana.*

Uma medida composta de desempenho foi definida como uma combinação de medidas primárias de desempenho, convertida em um único número para resumir múltiplas dimensões e facilitar comparações entre os centros. Um escore composto de um centro foi relatado como a média da medida composta do paciente a cada período de três meses.

### Análise estatística

Os dados são apresentados em frequência, média (desvio padrão), ou mediana (quartis). A avaliação do escore composto foi realizada usando um modelo linear de efeitos mistos com efeito do tempo polinomial (trimestre) e interceptos e inclinação aleatórios. Modelos com polinomiais de grau 1 a 5 foram justados e o melhor modelo usado com base nos critérios de Akaike. Componentes binários do último trimestre foram comparados com medidas basais usando a regressão logística mista, com intercepto aleatório ao centro.

O nível de significância foi estabelecido em 0,05 em todos os testes. O programa R (http://www.R-project.org) foi usado para as análises.

## Resultados

De março 2016 a novembro 2022, um total de 12167 pacientes com diagnóstico de SCA, IC, ou FA foram incluídos de 19 instituições de diferentes regiões do Brasil, principalmente nordeste e sudeste. Os dados demográficos e clínicos da população total de pacientes, que incluiu 2503 pacientes com FA, 3574 pacientes com IC, e 6090 pacientes com SCA estão apresentadas na Tabela 2. A idade mediana dos pacientes foi de 62,5 anos, e 61,1% eram homens. Havia uma alta prevalência de comorbidades, incluindo hipertensão (68,7%), diabetes mellitus (32,0%), e dislipidemia (24,1%).

**Tabela 2:** – Dados demográficos e história clínica dos pacientes

	FA (n=2503)	IC (n=3574)	SCA (n=6090)	Total (n=12167)
Idade; Mediana [IIQ]	66 [57,3 - 74,2]	61 [51 - 70,8]	61,7 [54,3-69,1]	62,5 [53,8 - 71]
Sexo masculino (%)	52,5	58,4	66,3	61,1
Raça branca (%)	49,7	31,5	38,4	38,7
Raça mulata (%)	36,9	52,2	47,7	46,7
Raça negra (%)	12,0	15,4	12,8	13,4
Raça amarela (%)	0,9	0,8	0,7	0,8
Hipertensão (%)	71,5	70,6	82,5	68,7
Diabetes mellitus (%)	24,1	35,1	41,5	32,0
Dislipidemia (%)	26,3	21,8	30,3	24,1
AVC prévio (%)	14,3	9,8	6,6	8,3
Doença arterial coronariana (%)	10,5	14	18	13,5
Doença arterial periférica (%)	4,9	3,8	2,5	3,1
Infarto do miocárdio (%)	11,7	18,7	20,7	16,2
Fibrilação atrial permanente / *Flutter* atrial (%)	81,9	25,4	3,2	24,8
Valvulopatia reumática (%)	6,6	6	0,3	3,1
Valvulopatia (%)	13,5	15,9	1,1	8,0
Diálise (%)	0,8	1,8	1,5	1,3
Insuficiência renal (Cr>2.0)(%)	5,4	14,6	3,6	6,6
Angioplastia prévia (%)	5,5	10,9	11,4	10
RM prévio (%)	4,2	5,7	3,4	4,2
Prótese valvar (%)	9,4	9,1	0,6	4,9
Tabagismo (%)	5,2	7,6	25,5	15,2
IMC, mediana [IIQ]	25,3 [21,9 - 28,9]	25,7 [22,2 - 29,6]	26,3 [23,7 - 29,3]	25,4 [22,3 - 28,9]
FEVE, mediana [IIQ]	61 [46 - 67]	36 [26 - 53]	54 [42 - 62]	51 [36 - 63]

*RM: revascularização do miocárdio; IMC: índice de massa corporal; FEVE: fração de ejeção do ventrículo esquerdo; AVC: acidente vascular cerebral; FA: fibrilação atrial; IC: insuficiência cardíaca; SCA: síndrome coronária aguda.*

**Figura 1:**
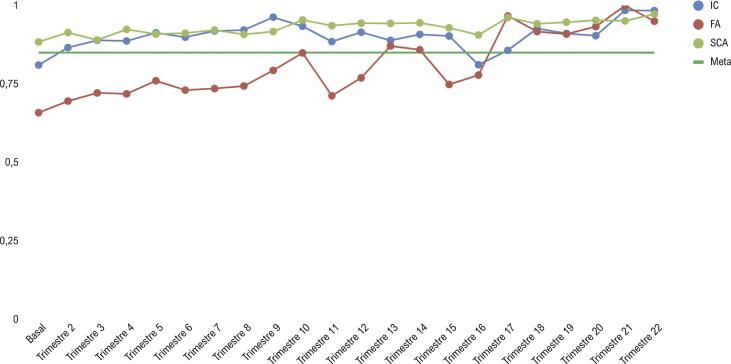
- Evolução das medidas compostas de desempenho a partir do basal ao longo do tempo (em trimestre); IC: insuficiência cardíaca; FA: fibrilação atrial; SCA: síndrome coronária aguda.

A Figura 1 apresenta os resultados das medidas compostas de desempenho no período de 22 trimestres de acompanhamento. Para considerar a variação do tempo em que os centros participantes estiveram envolvidos na coleta de dados, realizou-se uma análise por um período de 20 meses durante o qual o maior número de pacientes esteve presente (Tabela 3).

**Tabela 3:** – Medida composta de desempenho, durante um período de mais de 20 meses na maioria dos centros

Fibrilação atrial			
Trimestre	Valor médio	Diferença do basal	Valor p
Baseline	52,92 (42,53; 63,3)	-	-
Trimestre 2	59,24 (50,18; 68,31)	6,33 (2,93; 9,72)	< 0,001
Trimestre 3	63,83 (55,33; 72,32)	10,91 (4,93; 16,89)	< 0,001
Trimestre 4	66,66 (58,28; 75,04)	13,74 (5,69; 21,79)	< 0,001
Trimestre 5	67,74 (58,86; 76,62)	14,83 (4,76; 24,89)	0,004
Trimestre 6	67,08 (56,55; 77,61)	14,16 (1,59; 26,74)	0,027
**Insuficiência cardíaca**			
**Trimestre**	**Valor médio**	**Diferença do basal**	**Valor p**
Baseline	79,94 (74,78; 85,1)	-	-
Trimestre 2	83,02 (78,43; 87,61)	3,08 (1,09; 5,06)	0,002
Trimestre 3	85,24 (80,8; 89,69)	5,3 (2,1; 8,5)	0,001
Trimestre 4	86,61 (82,34; 90,88)	6,67 (2,88; 10,46)	< 0,001
Trimestre 5	87,12 (83,01; 91,24)	7,18 (3,08; 11,29)	< 0,001
Trimestre 6	86,78 (82,15; 91,41)	6,84 (2,02; 11,66)	0,005
**Síndrome coronária aguda**			
**Trimestre**	**Valor médio**	**Diferença do basal**	**Valor p**
Baseline	87,72 (83,89; 91,54)	-	-
Trimestre 2	87,31 (83,69; 90,94)	-0,4 (-2,72; 1,92)	0,734
Trimestre 3	88,16 (84,64; 91,69)	0,45 (-2,58; 3,47)	0,773
Trimestre 4	89,19 (85,44; 92,94)	1,48 (-2,27; 5,22)	0,44
Trimestre 5	89,34 (84,97; 93,71)	1,62 (-3,08; 6,32)	0,499
Trimestre 6	87,53 (82,36; 92,71)	-0,18 (-5,98; 5,61)	0,951

A Tabela 4 apresenta medidas de desempenho individuais para IC – uso de bloqueador de receptor de angiotensina / inibidor de enzima conversora de angiotensina (BRA/IECA) para pacientes com disfunção sistólica do ventrículo esquerdo (DSVE) e prescrição de antagonista de aldosterona – que apresentou melhora significativa do basal; para FA – acompanhamento do tempo de protrombina/ razão normalizada internacional (PT/INR), escore HAS-BLED e uso de estatina na alta hospitalar – que também mostrou melhora significativa; e para SCA – prescrição de aspirina na admissão hospitalar, betabloqueadores e estatina na alta. A Tabela 5 apresenta a mortalidade hospitalar, mortalidade em 180 dias, e novas internações.

**Tabela 4:** – Medidas de desempenho para fibrilação atrial, insuficiência cardíaca e síndrome coronária aguda

Medidas de desempenho para FA*	Baseline (n=364)	6 trimestres consecutivos (n=181)	p
BRA/IECA na alta	83,7	80,6	0,973
Escore CHADS-VASC2	47,4	43,1	0,461
Betabloqueadores na alta	81	91,8	0,101
Anticoagulação	78,4	82,8	0,318
Acompanhamento do TR/INR	87,1	97,7	0,002
Escore HAS-BLED	23,6	38,1	0,001
Estatina na alta	64,8	82,1	0,006
Escore médio composto	65,8 ± 36,2	73 ± 31,2	0,024
Medidas de desempenho para IC**	**Baseline (n=326)**	**6 trimestres consecutivos (n=224)**	**p**
BRA/IECA na alta para pacientes com DSVE	72,9	87,2	0,008
Betabloqueadores na alta para pacientes com DSVE	86,8	91,9	0,263
Avaliação da FEVE	90,8	94,9	0,128
Consulta pós-alta	82,5	88,8	0,077
Antagonista de aldosterona	64,2	82,8	0,004
Escore médio composto	81 ± 23,6	89,9 ± 19,3	<0,001
Medidas de desempenho para SCA***	**Baseline (n=485)**	**6 trimestres consecutivos (n=394)**	**p**
Aspirina na admissão hospitalar	90,2	94,7	0,035
Aspirina na alta	94,4	96,2	0,281
Betabloqueadores na alta	82,1	88,6	0,013
BRA/IECA na alta para pacientes com FEVE < 40%	79,6	83,8	0,546
Estatina na alta	87,3	95,2	<0,001
Controle da pressão arterial na alta	95,4	96,5	0,682
Aconselhamento ao adulto para cessação do tabagismo	92	75,3	0,004
Tempo porta-agulha	55,6	33,3	1
Tempo porta-balão	64,4	68,3%	0,621
Escore médio composto	88 ± 19,1	91,2 ± 14,9	<0,001

*Proporção de pacientes com fibrilação atrial (FA) não valvular/ pacientes com flutter atrial e avaliação do risco CHADS2-VASc documentada; proporção de pacientes com avaliação do risco HASBLED documentada; proporção de pacientes com fração de ejeção do ventrículo esquerdo (FEVE) < 40% ou pacientes com FA com FEVE ≤ 40% ou pacientes com síndrome coronária aguda (SCA) com FEVE < 45% e prescrição de inibidor de enzima conversora de angiotensina / bloqueador de receptor de angiotensina (IECA/BRA) na alta; proporção de pacientes com FA e alto risco de tromboembolismo de acordo com o CHADS2_VASc, tomando anticoagulantes; proporção de pacientes com FA com doença arterial coronariana, acidente vascular cerebral/ataque isquêmico transitório; doença vascular periférica ou diabetes, que receberam prescrição de estatina na alta; proporção de pacientes com SCA sem contraindicação e estatina prescrita para controle do colesterol LDL na alta; proporção de pacientes com FA usando varfarina e planejamento de acompanhamento da razão normalizada internacional (INR) na alta. **Proporção de pacientes com insuficiência cardíaca (IC) e disfunção ventricular esquerda documentada nos prontuários médicos ou em qualquer outro registro hospitalar acessível nos 12 meses antes da internação ou durante a internação, ou com plano de acompanhamento após a alta. Proporção de pacientes com IC e FEVE < 40% ou pacientes com FA e FEVE ≤ 40%, ou pacientes com SCA e FEVE < 45% com IECA/BRA prescrita na alta; proporção de pacientes com SCA e betabloqueador prescrito na alta; proporção de pacientes com IC e FEVE ≤ 35%, tomando inibidores de aldosterona; proporção de pacientes com IC com seguimento agendado e documentado. ***Proporção de pacientes com SCA recebendo aspirina nas 24 horas da internação no hospital; proporção de pacientes com SCA com prescrição de aspirina na alta, proporção de pacientes com SCA com prescrição de betabloqueador na alta; proporção de pacientes com IC e FEVE < 40% ou pacientes com FA com FEVE ≤ 40% ou pacientes com SCA e FEVE < 45% com IECA/BRA prescrito na alta; proporção de pacientes com SCA sem contraindicações e estatina prescrita para controle do colesterol LDL na alta; proporção de pacientes com SCA em uso de medicamento para controle da pressão arterial; proporção de pacientes com SCA que são fumantes ativos nos últimos 12 meses, recebendo aconselhamento para cessar o tabagismo durante a internação ou na alta; proporção de pacientes com Infarto Agudo do Miocárdio com supra de ST (IAMSST) submetidos à trombólise em 30 minutos, angioplastia primária em até 90 minutos após chegada no hospital IAMSST. IECA/BRA: inibidor de enzima conversora de angiotensina / bloqueador de receptor de angiotensina; FEVE: fração de ejeção do ventrículo esquerdo; DSVE: disfunção sistólica do ventrículo esquerdo; AVC: acidente vascular cerebral; AIT: ataque isquêmico transitório; TP/INR: tempo de protrombina/ razão normalizada internacional.

**Tabela 5:** – Mortalidade hospitalar, em 180 dias e novas hospitalizações

	Fibrilação atrial	Insuficiência cardíaca	Síndrome coronária aguda
**Mortalidade hospitalar**			
n/N (% [CI95%])	9/135 (6,67%)*	120/1305 (9,2%)	44/1959 (2,25%)
**Mortalidade**			
Taxa por 100 pacientes/ano (IC95%)	28/518 (5,4 [3,4 - 8,4])	146/440 (32,9 [27,0 - 40,1])	70/764 (9,1 [6,8 - 12,1])
**Internação**			
Taxa por 100 pacientes/ano (IC95%)	34/508 (6,5 [4,3 - 9,8])	189/389 (42,6 [35,8 - 50,7])	167/715 (21,7 [18,1 - 26,1])
**Internação ou morte**			
Taxa por 100 pacientes/ano (IC95%)	57/508 (10,9 [8,0 - 15,0])	312/389 (70,4 [61,5 - 80,5])	222/715 (28,9 [24,6 - 33,9])

*Intervalo de confiança para taxa estimada por regressão de Poisson, com tempo para o evento como offset.*

## Discussão

O Brasil apresenta um dos maiores sistemas de saúde público em termos de cobertura populacional, embora a qualidade da assistência oferecida pelo sistema seja frequentemente desafiada. A mortalidade hospitalar por doenças cardiovasculares no Brasil é ainda alta, e programas robustos de melhoria da qualidade são desejáveis e necessários. Programas como o GWTG mostraram serem capazes de melhorar o valor da assistência à saúde, identificando lacunas críticas, promovendo intervenções de melhoria de qualidade, medindo a taxa e o grau de mudança, e identificando o potencial para novas medidas de qualidade com base em resultados científicos.^6-9^

Desde seu início, a estratégia do programa BPC de 1) gerar novos conhecimentos, 2) identificar oportunidades de melhoria, 3) priorizar ações, e 4) implementar melhorias com base em evidência são valiosas para as instituições participantes. Uma vez identificadas a partir da análise dos indicadores, as intervenções propostas^2^ são coordenadas pelo grupo administrador do projeto. Elas incluem *checklists* e lembretes, *webinars*, relatórios automáticos e em tempo real por banco de dados eletrônico, materiais educativos, reuniões trimestrais para auditoria, *feedback* e reconhecimento, e treinamento de hospitais em metodologias para melhoria da qualidade para a implementação de ciclos rápidos de melhoria utilizando ferramentas promovidas pelo *Institute for Healthcare Improvement* que possibilitam que os hospitais desenvolvam planos de ação para alcançar a melhoria desejada.

Por ser um estudo de registro do mundo real, o programa BPC oferece uma informação abrangente do desempenho e da qualidade. Durante esse período, observamos uma melhoria geral a cada trimestre no cuidado baseado em evidência para FA, SCA e IC.

Hospitais universitários selecionados para o programa BPC tinham experiência prévia no manejo de paciente com IC. Isso explica seu melhor desempenho basal, com maior adesão às medidas de desempenho em comparação ao registro BREATHE.^10^ Embora isso represente um cenário favorável para os programas universitários, houve oportunidade para a melhoria de indicadores de desempenho como visto com a prescrição de BRA/IECA na alta hospitalar dos pacientes com DSVE e prescrição de antagonista de aldosterona pós-alta hospitalar. Um dado interessante foi que a prescrição de BRA/IECA na alta foi consideravelmente mais baixa em comparação ao programa GWTG.^6,7^ Observou-se boa adesão aos bloqueadores de receptor de aldosterona.

Em comparação ao estudo PEACE 5r-HF (*China Patient-centered evaluative Assessment of Cardiac Events Retrospective Study of Heart Failure*) conduzido na China,^11^ os pacientes do programa BPC eram mais jovens, tinham maior probabilidade de serem do sexo feminino, e apresentavam maiores taxas de hipertensão e diabetes.

Na alta hospitalar, as taxas de prescrição de IECAs e BRAs eram bem mais baixas na China (51,5% para IECAs ou BRAs) em comparação ao programa BPC (87,2%). Entre os candidatos elegíveis com IC e fração de ejeção reduzida, as taxas de prescrição de betabloqueadores foram de 46,2% na China e 91,9% no Brasil e de antagonistas de receptores de aldosterona foram 64,2% na alta na China em comparação a 82,8% no Brasil. A avaliação da Fração de Ejeção do Ventrículo Esquerdo (FEVE) é uma medida de qualidade fundamental no tratamento de pacientes com IC,^12^ e é notável que as instituições participantes do programa BPC obtêm índices similares aos de centros americanos e europeus. No entanto, o viés dos centros selecionados pode ter afetado esse resultado.^6,13^

O escore composto basal para FA no programa BPC foi bem similar ao relatado recentemente no estudo chinês.^14^ A hipertensão e o diabetes foram mais frequentes em nossa série. Como esperado, tivemos menos casos de novos diagnósticos de FA, uma vez que pacientes em tratamento ambulatorial também foram incluídos na análise.

Observou-se uma melhora marcante na determinação do escore HAS-BLED e na prescrição de estatina. Seguindo as recomendações de diretrizes,^15-17^ uma alta adesão à terapia com varfarina seria esperada, utilizando o TP/INR como controle do tratamento durante o acompanhamento.

Em relação à SCA, nossos dados diferiram-se aos do estudo chinês.^10^ Nossos pacientes eram mais jovens, e com uma maior proporção de mulheres. Hipertensão, diabetes, dislipidemia, e infarto do miocárdio prévio foram mais prevalentes em nossa série. Nossos resultados também foram diferentes de um registro nacional prévio conduzido no Brasil. Na população do nosso programa de BPC, tivemos mais pacientes com infarto do miocárdio que pacientes com angina instável.^18^

A taxa de prescrição de aspirina na alta foi de 96,2%, que é comparável a de diferentes países como o Reino Unido (98,1%) e a Suécia (94,6%).

Ainda, a taxa de uso de betabloqueadores na alta foi 88,6%, também comparável à do Reino Unido (95,6%) e da Suécia (88,7%).^19-21^

A comparação dos resultados do programa BPC com uma série do GWTG para SCA publicada,^6^ tivemos taxas similares de desempenho, superiores a 90% de adesão às medidas de desempenho. Uma exceção foram as taxas de uso de BRA/IECA na alta hospitalar para pacientes com FEVE <40% no BPC.

Em comparação ao projeto CCC-ACS da China,^22^ dados da SCA do programa BPC revelaram melhor desempenho, em termos de prescrição de aspirina, IECA/BRA, betabloqueadores e estatinas.

Também encontramos um aumento nas taxas de prescrição de betabloqueadores e estatinas na alta durante o estudo.

### Limitações

Há várias limitações do programa BPC que podem ter impacto sobre os resultados. A participação foi voluntária, e hospitais públicos terciários foram incluídos no estudo. Apesar de se haver planejado a coleta de dados de pacientes consecutivos, isso não foi sempre possível. Ainda, pelo fato de os serviços de emergência serem prestados em unidades de emergência no Brasil, não tivemos acesso a esses dados antes que o paciente chegasse no hospital. Por fim, mais estudos são necessários para avaliar medidas de desempenho e variância na qualidade entre os hospitais.

## Conclusões

O programa BPC é um programa de melhoria de qualidade no Brasil, em que dados em tempo real, derivados de diretrizes de cardiologia, foram implementados, com uma melhora global no manejo da FA, IC e SCA.

### Pesquisadores do programa BPC

*HCor* - Camila PP Toth, Camila RL Andretta, Denila B Silva, Erica DM Morosov, Patricia Vendramin, Suzana A Silva, Viviane B Campos; *Brazilian Society of Cardiology* - David Brasil, Fernanda Consolim, Fernando Bacal, Leandro I Zimerman; *HC-UFU- Uberlânda, MG*: Elmiro Resende (PI). Adriadne J Bertolin, Aguinaldo C Silva, Daniel B Oliveira ,Elaine F Silveiro, Fernanda R Souza, Poliana R Alves, Silvana F Andrade; *IC-DF- Brasília, DF*: Vitor S Barzilai (PI) Diego M Mesquita, Kaytiussia R Sena, Kenzo W Fernandes, Klícia BB Matioli, Rayane MC Lacourt, Ruanna M Rodrigues, Thaynara AS Silva; *Santa Lúcia Hospital - Poços de Caldas, MG*: Frederico TCD Orto (PI), Gislayne R Ribeiro, Luciana AP Andrade, Raquel Lopes, Ricardo R Bergo; *HC-UEL - Londrina, PR*: Manoel F Canesin (PI), Alessandra L Boçois, Daniela O Anjos, Fábio M Sekiyama, Fernando H Curan, Fabrício Furtado, Glaucia S S Maier, Juliana TM Lima, Lucas S Mello, Mayara CS Santos , Priscila B Ferreira, Vinícius AB Beleze; *Ana Nery Hospital - Salvador, BA*: Luiz CS Passos (PI), Marco AV Guedes (PI), Aline GB Jesus, Daniela C Dorta, Giedre A Ribeiro, Julia C Braga, Lucas H Oliveira, Marina B Martins, Osvaldo MS Neto, Ramana A Rangel, Rodrigo MV Melo, Rosane F Estevão, Tainara Cerqueira, Vitor C Fontes, William Carvalho; *HC-UFMG - Belo Horizonte, MG:* Luiz G Passaglia (PI), Ana CC Rios, Carolina T Cunha, Darkiane Ferreira, Gísia B Teodoro, Érika N Oliveira, Flávia M Mendes, Monique Rocha, Priscila T Paiva; *São Paulo Hospital/ UNIFESP - São Paulo, SP*: Andressa A Guerrero, Debora L Junqueira, Enia L Coutinho, Gabriela D Moura, Livia TC Bezerra, Lucia Coutinho; *Pedro Ernesto Hospital - UERJ - Rio de Janeiro, RJ*: Erika M Campana, Maria E Magalhães, Pedro Spineti, Simone Offrede; Rocio Hospital - Campo Largo, PR: Cesar O L Dusilek (PI), Daniele K Pokes. *Fundação HC Gaspar Vianna - Belém, PA*: Kleber RP Pereira (PI), Vitor BT Holanda (PI), Ana CAY Frazão, Christielaine V Zaninotto, Fabíola L Rolim, Fausto F Lobo, Louise SSV Boas, Luana S Freitas, Renata C Nunes, Rosana M Silva, Sheila Santos, Tácio SG Amoras, Yuri P Silva; *Messejana Hospital - Fortaleza, CE*: João DS Neto (PI), Dafne L Salles. Lia R Menezes, Lorena C Souza, Maria G V Sobral, Vera L Mendes, Viviane M Alves; *Santa Casa Macéio - Macéio, AL*: Maria A M Silva (PI), Flávia R S Araújo, Ivan R Rivera, Nayanne S Luz, Sávia N A Dórea, Valessa M A G Santana; *HC- UFPR - Curitiba, PR:* Miguel M F Silva (PI), Carolina R Senger, Eduardo L Adam, Gustavo S P Cunha, Jessica T Reichert, Karoline C Verka, Leonardo H S Melo, Lucas M Prado, Luiz G Matos, Niraj Mehta, Rafael Moretti; *HCPA - Porto Alegre, RS*: Mariana V Furtado (PI), Dayanna M P Lemos , Mauren P Haeffner, Letícia L Pedraza; *PROCAPE - Recife, PE:* Sérgio T Montenegro (PI), Dário C S Filho, Gabrielle P Silva, Karyne N Monte, Eveline L P Almeida, Suelen O Silva; *INC - Rio de Janeiro, RJ*: Marília Vasconcelos (PI), Tereza Felippe, Márcia R G Vasques, Glaucia R Silva, Robson M Nobre, Alexandre Vallado; *SCM Curitiba - Curitiba, PR:* Jose CM Jorge (PI); Angélica Chauchuti, Juliane Woehl, Marcelly G Bonatto, Mariana R Pius; H Com. Aeronáutica - Jaboatão dos Guararápes, PE: Rafel A F Gomes (PI), Breno Dantas; HC-UFMA - São Luis, MA: Jose AF Neto (PI), Adriana J Macau, Andre MS Figueiredo, Darci R Fernandes, Larissa A Lopes, Maria F Rocha, Renata M Assis, Tania PO Rocha, Willian M Penha; *HC-UFG - Goiânia, GO*: Weimar KS B Souza (PI), Camila D Pimenta, Diogo PS Sampaio, Heloisa BC Ribeiro, Natalia M Pereira, Marina M Siqueira, Murilo M Nunes, Rayne R Fagundes.

## References

[1] Moran AE, Forouzanfar MH, Roth GA, Mensah GA, Ezzati M, Murray CJ (2014). Temporal Trends in Ischemic Heart Disease Mortality in 21 World Regions, 1980 to 2010: The Global Burden of Disease 2010 Study. Circulation.

[2] Taniguchi FP, Bernardez-Pereira S, Silva SA, Ribeiro ALP, Morgan L, Curtis AB (2020). Implementation of a Best Practice in Cardiology (BPC) Program Adapted from Get with the Guidelines® in Brazilian Public Hospitals: Study Design and Rationale. Arq Bras Cardiol.

[3] Krumholz HM, Anderson JL, Bachelder BL, Fesmire FM, Fihn SD, Foody JM (2008). ACC/AHA 2008 Performance Measures for Adults with ST-Elevation and Non-ST-Elevation Myocardial Infarction: A Report of the American College of Cardiology/American Heart Association Task Force on Performance Measures (Writing Committee to Develop Performance Measures for ST-Elevation and Non-ST-Elevation Myocardial Infarction): Developed in Collaboration with the American Academy of Family Physicians and the American College of Emergency Physicians: Endorsed by the American Association of Cardiovascular and Pulmonary Rehabilitation, Society for Cardiovascular Angiography and Interventions, and Society of Hospital Medicine. Circulation.

[4] Schiele F, Gale CP, Bonnefoy E, Capuano F, Claeys MJ, Danchin N (2017). Quality Indicators for Acute Myocardial Infarction: A Position Paper of the Acute Cardiovascular Care Association. Eur Heart J Acute Cardiovasc Care.

[5] Jneid H, Addison D, Bhatt DL, Fonarow GC, Gokak S, Grady KL (2017). 2017 AHA/ACC Clinical Performance and Quality Measures for Adults with ST-Elevation and Non-ST-Elevation Myocardial Infarction: A Report of the American College of Cardiology/American Heart Association Task Force on Performance Measures. J Am Coll Cardiol.

[6] Ellrodt AG, Fonarow GC, Schwamm LH, Albert N, Bhatt DL, Cannon CP (2013). Synthesizing Lessons Learned from Get with the Guidelines: The Value of Disease-Based Registries in Improving Quality and Outcomes. Circulation.

[7] Cunningham LC, Fonarow GC, Yancy CW, Sheng S, Matsouaka RA, DeVore AD (2021). Regional Variations in Heart Failure Quality and Outcomes: Get with the Guidelines-Heart Failure Registry. J Am Heart Assoc.

[8] Smith SC, Fonarow GC, Zhao D (2020). Measuring and Improving the Quality of Heart Failure Care Globally. JAMA Netw Open.

[9] Peterson ED, Roe MT, Mulgund J, DeLong ER, Lytle BL, Brindis RG (2006). Association between Hospital Process Performance and Outcomes among Patients with Acute Coronary Syndromes. JAMA.

[10] Albuquerque DC, Souza JD, Bacal F, Rohde LE, Bernardez-Pereira S, Berwanger O (2015). I Brazilian Registry of Heart Failure - Clinical Aspects, Care Quality and Hospitalization Outcomes. Arq Bras Cardiol.

[11] Yu Y, Gupta A, Wu C, Masoudi FA, Du X, Zhang J (2019). Characteristics, Management, and Outcomes of Patients Hospitalized for Heart Failure in China: The China PEACE Retrospective Heart Failure Study. J Am Heart Assoc.

[12] Ponikowski P, Voors AA, Anker SD, Bueno H, Cleland JGF, Coats AJS (2016). 2016 ESC Guidelines for the Diagnosis and Treatment of Acute and Chronic Heart Failure: The Task Force for the Diagnosis and Treatment of Acute and Chronic Heart Failure of the European Society of Cardiology (ESC)Developed with the Special Contribution of the Heart Failure Association (HFA) of the ESC. Eur Heart J.

[13] Mozaffarian D, Benjamin EJ, Go AS, Arnett DK, Blaha MJ, Writing Group Members (2016). Heart Disease and Stroke Statistics-2016 Update: A Report from the American Heart Association. Circulation.

[14] Hao Y, Liu J, Smith SC, Huo Y, Fonarow GC, Ge J (2018). Rationale and Design of the Improving Care for Cardiovascular Disease in China (CCC) Project: A National Registry to Improve Management of Atrial Fibrillation. BMJ Open.

[15] Heidenreich PA, Estes NAM, Fonarow GC, Jurgens CY, Kittleson MM, Marine JE (2021). 2020 Update to the 2016 ACC/AHA Clinical Performance and Quality Measures for Adults with Atrial Fibrillation or Atrial Flutter: A Report of the American College of Cardiology/American Heart Association Task Force on Performance Measures. J Am Coll Cardiol.

[16] January CT, Wann LS, Calkins H, Chen LY, Cigarroa JE, Cleveland JC (2019). 2019 AHA/ACC/HRS Focused Update of the 2014 AHA/ACC/HRS Guideline for the Management of Patients with Atrial Fibrillation: A Report of the American College of Cardiology/American Heart Association Task Force on Clinical Practice Guidelines and the Heart Rhythm Society in Collaboration with the Society of Thoracic Surgeons. Circulation.

[17] Magalhães LP, Figueiredo MJO, Cintra FD, Saad EB, Kuniyoshi RR, Lorga AM (2016). Executive Summary of the II Brazilian Guidelines for Atrial Fibrillation. Arq Bras Cardiol.

[18] Piva e Mattos LA, Berwanger O, Santos ES, Reis HJ, Romano ER, Petriz JL (2013). Clinical Outcomes at 30 Days in the Brazilian Registry of Acute Coronary Syndromes (ACCEPT). Arq Bras Cardiol.

[19] Bradley EH, Herrin J, Elbel B, McNamara RL, Magid DJ, Nallamothu BK (2006). Hospital Quality for Acute Myocardial Infarction: Correlation Among Process Measures and Relationship with Short-Term Mortality. JAMA.

[20] Bebb O, Hall M, Fox KAA, Dondo TB, Timmis A, Bueno H (2017). Performance of Hospitals According to the ESC ACCA Quality Indicators and 30-day Mortality for Acute Myocardial Infarction: National Cohort Study Using the United Kingdom Myocardial Ischaemia National Audit Project (MINAP) Register. Eur Heart J.

[21] Chung SC, Gedeborg R, Nicholas O, James S, Jeppsson A, Wolfe C (2014). Acute Myocardial Infarction: A Comparison of Short-Term Survival in National Outcome Registries in Sweden and the UK. Lancet.

[22] Li J, Peng H, Zhao X, You N, Wu Y, Wang J (2020). Analysis of Situation of Acute Coronary Syndrome Based on the Date of the Improving Care for Cardiovascular Disease in China-Acute Coronary Syndrome (CCC-ACS) Project: Single-Centre Observational Study. Postgrad Med J.

