# Supplementary material for: Uma Iniciativa Nacional de Melhoria da Qualidade em Cardiologia: O Programa de Boas Práticas em Cardiologia no Brasil
Source: Arq Bras Cardiol. 2023 Nov 23;120(10):e20230375. [Article in Portuguese] doi: 10.36660/abc.20230375 (PMC10697684; doi:10.36660/abc.20230375)
Supplement: Translation

# Translation: A Nationwide Initiative to Improve Cardiology Quality: The Best Practice in Cardiology Program in Brazil

**Authors:** Fabio Papa Taniguchi, Sabrina Bernardez-Pereira, Antônio Luiz Pinho Ribeiro, Louise Morgan, Anne B. Curtis, Kathryn Taubert, Denilson Campos de Albuquerque, Sidney C Smith, Angêlo Amato Vincenzo de Paola

**Affiliations:** Brazil Hospital do Coração,1 São Paulo, SP – Brazil; Brazil Universidade Federal de Minas Gerais,2 Belo Horizonte, MG – Brazil; USA American Heart Association Inc,3 Dallas, Texas – USA; USA University at Buffalo – The State University of New York,4 Buffalo, New York – USA; Switzerland American Heart Association,5 Basel – Switzerland; Brazil Universidade do Estado do Rio de Janeiro,6 Rio de Janeiro, RJ – Brazil; USA University of North Carolina at Chapel Hill,7 Chapel Hill, North Carolina – USA; Brazil Escola Paulista de Medicina da Universidade Federal de São Paulo – UNIFESP,8 São Paulo, SP – Brazil

**Keywords:** Cardiology, Quality Improvement, Evidence-Based Practice

## Abstract

**Background:**

Despite significant progress in improving the quality of cardiovascular care, persistent gaps remain in terms of inconsistent adherence to guideline recommendations.

**Objective:**

This study evaluates the effects of implementing a quality improvement program adapted from the American Heart Association’s Get with the Guidelines™ initiative on adherence to guideline-directed medical therapy for acute coronary syndrome (ACS), atrial fibrillation (AF), and heart failure (HF).

**Methods:**

We examined demographics, quality measures, and short-term outcomes in patients hospitalized with ACS, AF, and HF enrolled in the Best Practice in Cardiology (BPC) Program from 2016 to 2022.

**Results:**

This study included 12,167 patients in 19 hospitals in Brazil. Mean age was 62.5 [53.8-71] y/o; 61.1% were male, 68.7% had hypertension, 32.0% diabetes mellitus, and 24.1% had dyslipidemia. Composite score had a sustainable performance in the period from baseline to the last quarter: 65.8±36.2% to 73± 31.2% for AF (p=0.024), 81.0± 23.6% to 89.9 ± 19.3% for HF (p<0.001), and from 88.0 ± 19.1 to 91.2 ±14.9 for ACS (p<0.001).

**Conclusions:**

The BPC program is a quality improvement program in Brazil in which real-time data, obtained using cardiology guideline metrics, were implemented in a quality improvement program resulting in an overall sustained improvement in AF, HF, and ACS management.

**Central Illustration:** : A Nationwide Initiative to Improve Cardiology Quality: The Best Practice in Cardiology Program in Brazil

*ACEI/ARB: angiotensin-converting enzyme inhibitor/angiotensin receptor blocker; LVEF: left ventricular ejection fraction; LVSD: left ventricular systolic dysfunction; AF: atrial fibrillation; PT/INR: prothrombin time/international normalized ratio.*

## Introduction

Cardiovascular disease (CVD) imposes significant health and economic burdens in Brazil, and the country has one of the highest mortality rates for CVD globally, comparable to China and Eastern Europe.^1^ To address this issue, the Best Practice in Cardiology (BPC) program was implemented in Brazil. Collaboratively led by Hospital do Coração (HCor), the Brazilian Society of Cardiology (SBC), the American Heart Association (AHA), and the Ministry of Health, the BPC program aims to improve CVD care by adapting quality improvement programs from Get with the Guidelines (GWTG)™.^2-6^

Notably, the BPC program in Brazil is the first instance of simultaneously initiating three quality programs adapted from the AHA outside the USA. Specifically, the program focuses on enhancing care for acute coronary syndrome (ACS), heart failure (HF), and atrial fibrillation (AF). Its objectives include (1) describing the characteristics, in-hospital treatment, and outcomes of patients admitted to public hospitals in Brazil, (2) evaluating the effectiveness of quality improvement programs in enhancing care quality and outcomes, and (3) exploring and optimizing quality improvement strategies within the Brazilian healthcare system.

This article presents the outcomes and findings of the BPC program, aiming to identify additional opportunities for quality improvement and provide guidance for the development of effective strategies and tools to improve CVD outcomes in Brazil.

## Methods

### Study design

The BPC program is a quality improvement initiative with a nationwide registry that focuses on quality-of-care measures for ACS, HF, and AF (Central Figure). A standard procedure was used during data collection from the patients’ medical records and regular quality audits to ensure the accuracy and completeness of research data were performed by the coordination center. Institutional review board approval was granted for this research by the ethics committee of HCor, São Paulo, Brazil, number 48561715.5.1001.0060, and of each participating hospital. Details of the study design and methodology of the BPC program have been described elsewhere.^2^

Data collected included demographic variables, prehospital information, current medical assessment in the hospital, medical history and risk factors, current hospitalization diagnosis and treatment, drug treatment, clinical events during hospitalization, discharge guidance, and discharge diagnosis.

### Outcome measures

Primary performance measures were designed to evaluate the quality of care for patients with ACS, HF, and AF. They were developed according to the Brazilian Society of Cardiology and American College of Cardiology /American Heart Association statements and guidelines. Performance measures for each critical condition were analyzed for each center before and after its participation in the BPC program (Table 1).

**Table 1:** – Performance measures

Atrial Fibrillation
ARB/ACEI Prescribed Prior to Discharge (When LVEF ≤40)
CHA2DS2–VASc risk Score Documented Prior to Discharge
β-blocker Prescribed Prior to Discharge (when LVEF ≤ 40)
Anticoagulation Prescription Prior to Discharge
PT/INR Planned Follow-Up Documented Prior to Discharge for Warfarin TreatmenT
HAS-BLED risk Score Documented Prior to Discharge
Statin at discharge in AF patients with CAD, CVA/TIA, PVD, or Diabetes
**Acute coronary syndrome**
Aspirin within 24 hours of admission
Aspirin at discharge
β-blockers at discharge
ARB/ACEI Prescribed Prior to Discharge (When LVEF ≤40)
Statin at discharge
Adult smoking cessation advice/counseling
High Blood Pressure control
30 - Minute Door- to - Needle Time
90 -Minute Door-to- Balloon Time
**Heart Failure**
ARB/ACEI at discharge for patients with LVSD
β-blockers at discharge for patients with LVSD, no contraindications
LVEF assessment
Post-discharge appointment
Aldosterone antagonist prescription

*ARB/ACEI: angiotensin receptor blocker/angiotensin-converting enzyme inhibitor; GWTG-HF: Get With The Guidelines-Heart Failure; LVEF: left ventricular ejection fraction; LVSD: left ventricular systolic dysfunction; CVA: cerebrovascular accident; TIA: transient ischemic attack; PVD: peripheral vascular disease; AF: atrial fibrillation; CAD: coronary artery disease.*

A composite performance measure was defined by the combination of primary performance measures, which was converted into a single number to summarize multiple dimensions and facilitate comparisons among the centers. A center’s composite score was reported as the patient’s mean composite measure in each three-month period.

### Statistical analysis

Data are presented as frequencies, mean (standard deviation), or median (quartiles). Assessment of the composite score was performed using a linear mixed-effect model with polynomial time (quarters) effect and random intercepts and slope. Models with 1 to 5 degree polynomials were adjusted and the best model was used on the basis of Akaike’s criteria. Binary components of the last quarter available were compared with baseline measures using mixed logistic regression with random intercept by the center.

The significance level was set at 0.05 for all tests. R software (http://www.R-project.org) was used for all statistical analyses.

## Results

From March 2016 to November 2022, a total of 12,167 patients with a diagnosis of ACS, HF, or AF were enrolled at 19 institutions located in different regions of Brazil, mostly the northeast and southeast regions. Demographic and clinical data of the patient population, which included 2,503 AF patients, 3,574 HF patients, and 6,090 ACS patients, are presented in Table 2. The median age of the patient population was 62.5 years, and 61.1% were men. There was a high prevalence of comorbidities, including hypertension (68.7%), diabetes mellitus (32%), and dyslipidemia (24.1%) in the patient population.

**Table 2:** – Patient demographics and past medical history

	AF (n=2,503)	HF (n=3,574)	ACS(n=6090)	Total (n=12,167)
Age; median [IQR]	66 [57.3 - 74.2]	61 [51 - 70.8]	61.7 [54.3-69.1]	62.5 [53.8 - 71]
Gender; male (%)	52.5	58.4	66.3	61.1
Race; White (%)	49.7	31.5	38.4	38.7
Race; Brown (%)	36.9	52.2	47.7	46.7
Race; Black (%)	12.0	15.4	12.8	13.4
Race; Asian (%)	0.9	0.8	0.7	0.8
Hypertension (%)	71.5	70.6	82.5	68.7
Diabetes mellitus (%)	24.1	35.1	41.5	32.0
Dyslipidemia (%)	26.3	21.8	30.3	24.1
Previous stroke (%)	14.3	9.8	6.6	8.3
Coronary artery disease (%)	10.5	14	18	13.5
Peripheral artery disease (%)	4.9	3.8	2.5	3.1
Myocardial Infarction (%)	11.7	18.7	20.7	16.2
Permanent Atrial Fibrillation / Atrial flutter (%)	81.9	25.4	3.2	24.8
Rheumatic valvular disease (%)	6.6	6	0.3	3.1
Valvular disease (%)	13.5	15.9	1.1	8.0
Dialysis (%)	0.8	1.8	1.5	1.3
Kidney failure (Cr>2.0)(%)	5.4	14.6	3.6	6.6
Previous angioplasty (%)	5.5	10.9	11.4	10
Previous CABG (%)	4.2	5.7	3.4	4.2
Valvular prosthesis (%)	9.4	9.1	0.6	4.9
Current smoking (%)	5.2	7.6	25.5	15.2
BMI, median [IQR]	25.3 [21.9 - 28.9]	25.7 [22.2 - 29.6]	26.3 [23.7 - 29.3]	25.4 [22.3 - 28.9]
LVEF, median [IQR]	61 [46 - 67]	36 [26 - 53]	54 [42 - 62]	51 [36 - 63]

*CABG: coronary artery bypass grafting; BMI: body mass index; LVEF: left ventricular ejection fraction; IQR: interquartile range; AF: atrial fibrillation; HF: heart failure; ACS: acute coronary syndrome.*

Figure 1 presents the results for the compound performance measures over 22 quarters of follow-up. To account for the variation in the length of time that participating centers were involved in data collection, an analysis was conducted over a 20-month period during which the highest number of patients was present (Table 3).

**Figure 1:** -Changes of composite performance measures from baseline over time (in quarters); HF: heart failure; AF: atrial fibrillation; ACS: acute coronary syndrome.

**Table 3:** – Composite performance measure over a 20-month period in most centers

Atrial fibrillation			
Quarter	Mean Value	Difference from baseline	p value
Baseline	52.92 (42.53; 63.3)	-	-
Quarter 2	59.24 (50.18; 68.31)	6.33 (2.93; 9.72)	< 0.001
Quarter 3	63.83 (55.33; 72.32)	10.91 (4.93; 16.89)	< 0.001
Quarter 4	66.66 (58.28; 75.04)	13.74 (5.69; 21.79)	< 0.001
Quarter 5	67.74 (58.86; 76.62)	14.83 (4.76; 24.89)	0.004
Quarter 6	67.08 (56.55; 77.61)	14.16 (1.59; 26.74)	0.027
**Heart Failure**			
**Quarter**	**Mean Value**	**Difference from baseline**	**p value**
Baseline	79.94 (74.78; 85.1)	-	-
Quarter 2	83.02 (78.43; 87.61)	3.08 (1.09; 5.06)	0.002
Quarter 3	85.24 (80.8; 89.69)	5.3 (2.1; 8.5)	0.001
Quarter 4	86.61 (82.34; 90.88)	6.67 (2.88; 10.46)	< 0.001
Quarter 5	87.12 (83.01; 91.24)	7.18 (3.08; 11.29)	< 0.001
Quarter 6	86.78 (82.15; 91.41)	6.84 (2.02; 11.66)	0.005
**Acute Coronary Syndrome**			
**Quarter**	**Mean Value**	**Difference from baseline**	**p value**
Baseline	87.72 (83.89; 91.54)	-	-
Quarter 2	87.31 (83.69; 90.94)	-0.4 (-2.72; 1.92)	0.734
Quarter 3	88.16 (84.64; 91.69)	0.45 (-2.58; 3.47)	0.773
Quarter 4	89.19 (85.44; 92.94)	1.48 (-2.27; 5.22)	0.44
Quarter 5	89.34 (84.97; 93.71)	1.62 (-3.08; 6.32)	0.499
Quarter 6	87,53 (82,36; 92,71)	-0,18 (-5,98; 5,61)	0,951

Table 4 presents individual performance measures for HF – angiotensin receptor blocker/angiotensin-converting enzyme inhibitor (ARB/ACEI) use at discharge for patients with left ventricular systolic dysfunction (LVSD) and aldosterone antagonist prescription – which had significant improvement from baseline; for AF – prothrombin time/international normalized ratio (PT/INR) planned follow-up, HAS-BLED score, and statin at discharge), which also had significant improvement; and for ACS - aspirin within 24 hours of admission, beta-blockers and statin at discharge had significant improvement.

**Table 4:** – Performance measures for AF, HF and ACS

Performance measures for AF*	Baseline (n=364)	at 6 continuos quarters (n=181)	p
ARB/ACEI at discharge	83.7	80.6	0.973
CHADS-VASC2 score	47.4	43.1	0.461
β-Blockers at discharge	81	91.8	0.101
Anticoagulation	78.4	82.8	0.318
PT/INR Planned Follow-Up	87.1	97.7	0.002
HAS-BLED score	23.6	38.1	0.001
Statin at discharge	64.8	82.1	0.006
Composite score	65.8 ± 36.2	73 ± 31.2	0.024
Performance Measures for HF**	**Baseline (n=326)**	**at 6 continuous quarters (n=224)**	**p**
ARB/ACEI at discharge for patients with LVSD	72.9	87.2	0.008
β-Blockers at discharge for patients with LVSD	86.8	91.9	0.263
LVEF assessment	90.8	94.9	0.128
Postdischarge appointment	82.5	88.8	0.077
Aldosterone Antagosnist	64.2	82.8	0.004
Composite score	81 ± 23.6	89.9 ± 19.3	<0.001
Performance Measures for ACS***	**Baseline (n=485)**	**at 6 continuous quarters (n=394)**	**p**
Aspirin within 24 h of admission	90.2	94.7	0.035
Aspirin at discharge	94.4	96.2	0.281
β-Blockers at discharge	82.1	88.6	0.013
ARB/ACEI at discharge for patients with LVEF < 40%	79.6	83.8	0.546
Statin at discharge	87.3	95.2	<0,001
Blood pressure control at discharge	95.4	96.5	0.682
Adult smoking cessation advice/counseling	92	75.3	0.004
Door-to-needle time	55.6	33.3	1
Door-to-balloon time	64.4	68.3%	0.621
Composite score	88 ± 19.1	91.2 ± 14.9	<0.001

*Proportion of non-valvular AF/Flutter patients with a documented CHADS2-VASc risk score assessment; Proportion of patient with a documented HAS-BLED risk score assessment; Proportion of HF patients with LVEF < 40% or AF patients with LVEF ≤ 40% or ACS patients with LVEF < 45% with an ACEI/ARB prescribed at discharge; Proportion of ACS patients with a beta blocker prescribed at discharge; Proportion of AF patients at high risk for thromboembolism according to the CHADS2_VASc taking anticoagulants; Proportion of AF patients with CAD, stroke/TIA, PVD or diabetes who were prescribed a statin at discharge; Proportion of ACS patients without contraindications with statin prescribed for LDL control at discharge; Proportion of AF patients discharged on Warfarin who had an INR follow up planned prior to hospital discharge. **Proportion of HF patients with a documented LV dysfunction either in the medical records or other reports accessible in hospital charts in the 12 months before admission or during hospitalization or with a scheduled evaluation planned to be performed after discharge; Proportion of HF patients with LVEF < 40% or AF patients with LVEF ≤ 40% or ACS patients with LVEF < 45% with an ACEI/ARB prescribed at discharge; Proportion of ACS patients with a beta blocker prescribed at discharge; Proportion of HF patients with LVEF ≤ 35% taking aldosterone inhibitors; Proportion of HF patients for whom a follow-up appointment was scheduled and documented. ***Proportion of ACS patients receiving aspirin within 24 hours of hospital arrival; Proportion of ACS patients with aspirin prescribed at discharge; Proportion of ACS patients with a beta blocker prescribed at discharge; Proportion of HF patients with LVEF < 40% or AF patients with LVEF ≤ 40% or ACS patients with LVEF < 45% with an ACEI/ARB prescribed at discharge; Proportion of ACS patients without contraindications with statin prescribed for LDL control at discharge; Proportion of ACS patients under medication for blood pressure control; Proportion of ACS patients, who are active smoker within the past 12 months, who receive smoking cessation advice during hospitalization or at discharge; Proportion of STEAMI patients submitted to thrombolysis within 30 min; Proportion of STEAMI patients submitted to primary angioplasty within 90 min from hospital arrival.

Table 5 presents in-hospital mortality, mortality at 180 days, and new hospitalizations.

**Table 5:** – In-hospital mortality, mortality at 180 days and new hospitalizations

	Atrial fibrillation	Heart failure	Acute coronary syndrome
**Hospital mortality**			
n/N (% [CI95%])	9/135 (6.67%)*	120/1305 (9.2%)	44/1959 (2.25%)
**Mortality**			
Rate per 100 patient-year (95%CI)	28/518 (5.4 [3.4 - 8.4])	146/440 (32.9 [27.0 - 40.1])	70/764 (9.1 [6.8 - 12.1])
**Hospitalization**			
Rate per 100 patient-year (95%CI)	34/508 (6.5 [4.3 - 9.8])	189/389 (42.6 [35.8 - 50.7])	167/715 (21.7 [18.1 - 26.1])
**Hospitalization or death**			
Rate per 100 patient-year (95%CI)	57/508 (10.9 [8.0 - 15.0])	312/389 (70.4 [61.5 - 80.5])	222/715 (28.9 [24.6 - 33.9])

*Confidence interval for Rate estimated by Poisson regression with time to event as offset.*

## Discussion

Brazil has one of the largest publicly financed patient care systems regarding population coverage, although the quality of assistance provided by the public system is frequently challenged. Cardiovascular in-hospital mortality in Brazil is still high, and well-designed, robust quality improvement programs are desirable and needed. Programs like GWTG have shown to improve healthcare value by identifying critical gaps, promoting quality improvement interventions, measuring the rate and degree of change, and identifying potential for new quality measures based on evolving scientific results.^6-9^

Since its beginning, the strategy of the BPC program to 1) generate new knowledge, 2) identify opportunities for improvement, 3) prioritize actions and 4) implement improvements based on evidence has been an asset to the participating institutions. Once identified from the analysis of the indicators, the proposed interventions^2^ are centrally coordinated by the project management group. They include checklists and reminders, webinars, automatic and real-time reports through an electronic database, educational materials, quarterly meetings for audit, feedback and recognition, and training of hospitals in quality improvement methodologies for the implementation of rapid improvement cycles using tools promoted by the Institute for Healthcare Improvement to enable hospitals to develop action plans to achieve the desired improvement.

As a real-world registry study, the BPC program provides comprehensive performance and quality information. During this period, we observed an overall sustained improvement from quarter to quarter in evidence-based care for AF, ACS, and HF.

Selected university hospitals for the BPC program had previous experience in the management of patients with HF. This explains their better baseline performance, with higher adherence to performance measures compared to the BREATHE Registry.^10^ Although this represents a favorable scenario for the university programs, there was room for quality improvement as seen in ARB/ACEI at discharge for patients with LVSD and aldosterone antagonist prescription. Interestingly, ARB/ACEI at discharge was remarkably low during the period compared to the GWTG program.^6,7^ For the aldosterone receptor blockers prescription, a life-saving medication, there was a marked adherence in its use.

Compared with the PEACE 5r-HF (China Patient-centered evaluative Assessment of Cardiac Events Retrospective Study of Heart Failure) in China^11^ the BPC program patients were younger, more likely to be female and had higher rates of high blood pressure and diabetes.

At discharge, prescription rates of ACEIs and ARBs were much lower in China (51.5% for either ACEIs or ARBs) compared to the BPC program (87.2%). Among eligible candidates with HF with reduced ejection fraction, prescription rates of beta-blockers were 46.2% in China and 91.9% in Brazil and aldosterone receptor antagonists were 64.2% at discharge in China compared to 82.8% in Brazil.

The evaluation of left ventricular ejection fraction (LVEF) is a fundamental measure of quality for treating patients with HF,^12^ and it is noticeable that the BPC program institutions obtain indices similar to those of American and European centers. However, note that the bias of the selected centers may have affected this result.^6,13^

The composite baseline score for AF in the BPC program was quite similar to the recent report of the Chinese experience.^14^ Hypertension and diabetes were more frequent in our series. As expected, we had fewer cases of newly diagnosed AF as outpatients were also included in the analysis.

There was marked improvement in HAS-BLED score determination and statin prescription. According to guideline recommendations,^15-17^high adherence to warfarin therapy would be expected using PT/INR during follow-up for treatment control.

Regarding ACS, our data differed from the Chinese study.^10^ Our patients were younger, and with a greater proportion of female. Hypertension, diabetes, dyslipidemia, and previous myocardial infarction were more prevalent in our series. Our results also differ from a previous national registry in Brazil. In our BPC program population, we had more patients with myocardial infarction rather than unstable angina.^18^

The prescription rate of aspirin at discharge was 96.2%, which is comparable to different countries like the United Kingdom (98.1%) and Sweden (94.6%).

Also, beta-blockers at discharge from the hospital was 88.6%, also comparable with the United Kingdom (95.6%) and Sweden (88.7%).^19-21^

Comparing the BPC results with a published series of GWTG for ACS,^6^ we had similar performance rates, over 90% adherence to the performance measures. One exception was ARB/ACEI rates at discharge for patients with LVEF < 40% in the BPC.

Compared to the CCC-ACS project from China,^22^ ACS data from the BPC program revealed better performance, in terms of prescription of aspirin, ACEI/ARB, beta-blockers, and statins.

For ACS, we found an increase in the prescription ratesof aspirin within 24 h of admission, beta-blockers and statins at discharge.

### Limitations

There are several limitations of the BPC program that may impact the results. Participation was voluntary, and public tertiary hospitals were enrolled in this study. Although intended to collect consecutive patient data, this was not always possible. Also, as emergency services are provided at emergency care units in Brazil, we did not have access to these data before the patient’s arrival at the hospital. Finally, further studies are needed to assess performance measures and variance in quality across hospitals.

## Conclusions

The BPC program is a quality improvement program in Brazil in which real-time data, derived from cardiology guidelines, were implemented with an overall sustained improvement in AF, HF, and ACS management.

### The BPC Investigators

*HCor* - Camila PP Toth, Camila RL Andretta, Denila B Silva, Erica DM Morosov, Patricia Vendramin, Suzana A Silva, Viviane B Campos; *Brazilian Society of Cardiology* - David Brasil, Fernanda Consolim, Fernando Bacal, Leandro I Zimerman; *HC-UFU- Uberlânda, MG*: Elmiro Resende (PI). Adriadne J Bertolin, Aguinaldo C Silva, Daniel B Oliveira ,Elaine F Silveiro, Fernanda R Souza, Poliana R Alves, Silvana F Andrade; *IC-DF- Brasília, DF*: Vitor S Barzilai (PI) Diego M Mesquita, Kaytiussia R Sena, Kenzo W Fernandes, Klícia BB Matioli, Rayane MC Lacourt, Ruanna M Rodrigues, Thaynara AS Silva; *Santa Lúcia Hospital - Poços de Caldas, MG*: Frederico TCD Orto (PI), Gislayne R Ribeiro, Luciana AP Andrade, Raquel Lopes, Ricardo R Bergo; *HC-UEL - Londrina, PR*: Manoel F Canesin (PI), Alessandra L Boçois, Daniela O Anjos, Fábio M Sekiyama, Fernando H Curan, Fabrício Furtado, Glaucia S S Maier, Juliana TM Lima, Lucas S Mello, Mayara CS Santos , Priscila B Ferreira, Vinícius AB Beleze; *Ana Nery Hospital - Salvador, BA*: Luiz CS Passos (PI), Marco AV Guedes (PI), Aline GB Jesus, Daniela C Dorta, Giedre A Ribeiro, Julia C Braga, Lucas H Oliveira, Marina B Martins, Osvaldo MS Neto, Ramana A Rangel, Rodrigo MV Melo, Rosane F Estevão, Tainara Cerqueira, Vitor C Fontes, William Carvalho; *HC-UFMG - Belo Horizonte, MG:* Luiz G Passaglia (PI), Ana CC Rios, Carolina T Cunha, Darkiane Ferreira, Gísia B Teodoro, Érika N Oliveira, Flávia M Mendes, Monique Rocha, Priscila T Paiva; *São Paulo Hospital/ UNIFESP - São Paulo, SP*: Andressa A Guerrero, Debora L Junqueira, Enia L Coutinho, Gabriela D Moura, Livia TC Bezerra, Lucia Coutinho; *Pedro Ernesto Hospital - UERJ - Rio de Janeiro, RJ*: Erika M Campana, Maria E Magalhães, Pedro Spineti, Simone Offrede; Rocio Hospital - Campo Largo, PR: Cesar O L Dusilek (PI), Daniele K Pokes. *Fundação HC Gaspar Vianna - Belém, PA*: Kleber RP Pereira (PI), Vitor BT Holanda (PI), Ana CAY Frazão, Christielaine V Zaninotto, Fabíola L Rolim, Fausto F Lobo, Louise SSV Boas, Luana S Freitas, Renata C Nunes, Rosana M Silva, Sheila Santos, Tácio SG Amoras, Yuri P Silva; *Messejana Hospital - Fortaleza, CE*: João DS Neto (PI), Dafne L Salles. Lia R Menezes, Lorena C Souza, Maria G V Sobral, Vera L Mendes, Viviane M Alves; *Santa Casa Macéio - Macéio, AL*: Maria A M Silva (PI), Flávia R S Araújo, Ivan R Rivera, Nayanne S Luz, Sávia N A Dórea, Valessa M A G Santana; *HC- UFPR - Curitiba, PR:* Miguel M F Silva (PI), Carolina R Senger, Eduardo L Adam, Gustavo S P Cunha, Jessica T Reichert, Karoline C Verka, Leonardo H S Melo, Lucas M Prado, Luiz G Matos, Niraj Mehta, Rafael Moretti; *HCPA - Porto Alegre, RS*: Mariana V Furtado (PI), Dayanna M P Lemos , Mauren P Haeffner, Letícia L Pedraza; *PROCAPE - Recife, PE:* Sérgio T Montenegro (PI), Dário C S Filho, Gabrielle P Silva, Karyne N Monte, Eveline L P Almeida, Suelen O Silva; *INC - Rio de Janeiro, RJ*: Marília Vasconcelos (PI), Tereza Felippe, Márcia R G Vasques, Glaucia R Silva, Robson M Nobre, Alexandre Vallado; *SCM Curitiba - Curitiba, PR:* Jose CM Jorge (PI); Angélica Chauchuti, Juliane Woehl, Marcelly G Bonatto, Mariana R Pius; H Com. Aeronáutica - Jaboatão dos Guararápes, PE: Rafel A F Gomes (PI), Breno Dantas; HC-UFMA - São Luis, MA: Jose AF Neto (PI), Adriana J Macau, Andre MS Figueiredo, Darci R Fernandes, Larissa A Lopes, Maria F Rocha, Renata M Assis, Tania PO Rocha, Willian M Penha; *HC-UFG - Goiânia, GO*: Weimar KS B Souza (PI), Camila D Pimenta, Diogo PS Sampaio, Heloisa BC Ribeiro, Natalia M Pereira, Marina M Siqueira, Murilo M Nunes, Rayne R Fagundes.
